# Functional analysis of the short splicing variant encoded by CHI3L1/YKL-40 in glioblastoma

**DOI:** 10.3389/fonc.2022.910728

**Published:** 2022-11-02

**Authors:** Mengqi Shi, Qianyun Ge, Xinrong Wang, Wenbin Diao, Ben Yang, Sipeng Sun, Guohui Wang, Tian Liu, Andrew Man-Lok Chan, Zhiqin Gao, Yi Wang, Yubing Wang

**Affiliations:** ^1^ School of Life Science and Technology, Weifang Medical University, Weifang, China; ^2^ Community Healthcare Center, The Second People’s Hospital of Weifang, Weifang, China; ^3^ School of Biomedical Sciences, The Chinese University of Hong Kong, Hong Kong, Hong Kong SAR, China

**Keywords:** YKL-40, splicing variant, glioblastoma, transcriptomes, protein secretion

## Abstract

The glycoprotein YKL-40 has been well studied as a serum biomarker of prognosis and disease status in glioblastoma. YKL-40 is a chitinase-like protein with defective chitinase activity that plays an important role in promoting cell proliferation, migration, and metastasis in glioblastoma multiforme (GBM). The short variant (SV) of YKL-40, generated by an alternative splicing event that splices out exon 8, was reported in the early developing human musculoskeletal system, although its role in GBM is still unknown. Our results showed that individual glioblastoma cell lines displayed increased expression of the short variant of YKL-40 after low serum treatment. In addition, unlike the full-length (FL) version, which was localized to all cell compartments, the short isoform could not be secreted and was localized only to the cytoplasm. Functionally, FL YKL-40 promoted cell proliferation and migration, whereas SV YKL-40 suppressed them. Transcriptome analysis revealed that these opposing roles of the two isoforms may be modulated by differentially regulating several oncogenic-related pathways, including p53, the G2/M checkpoint, and MYC-related signaling. This study may provide new ideas for the development of targeted anti-YKL-40 therapy in GBM treatment.

## Introduction

Glioblastoma multiforme (GBM), also classified as grade IV astrocytoma, represents the most lethal type of brain tumor, with a median overall survival of 12–16 months (1). GBM progresses rapidly, with a median survival of 13 months after diagnosis and a 5-year survival rate of less than 5%. Using the gene expression datasets of a large group of GBM patients, researchers working on The Cancer Genome Atlas (TCGA) identified four different molecular subtypes of GBM: proneural, neural, classical, and mesenchymal (2). Among them, the mesenchymal subtype is the most consistently described in the literature, representing up to 34% of GBM samples (3). Mesenchymal tumors are significantly associated with poor radiation response and worse survival. Approximately 30% of proneural tumors shift to the mesenchymal signature upon recurrence, marked by loss of expression of the proneural marker OLIG2 and upregulation of mesenchymal gene YKL-40 (4). The proneural–mesenchymal transition, similar to the epithelial–mesenchymal transition (EMT) process in other cancers, plays an essential role in tumor cell migration, metastasis, and therapeutic resistance (5). Therefore, understanding the fundamental role of mesenchymal markers in regulating tumor progression may be key to developing more effective therapeutic interventions in GBM.

YKL-40 is a secreted glycoprotein that lacks glycosyl hydrolase activity and is encoded by the chitinase-3-like-1 gene (*CHI3L1*) (6). YKL-40 has been defined as a marker for the mesenchymal subtype of GBM (7) and plays an important role in promoting angiogenesis, cell proliferation, cell survival, and invasion (8, 9). Increased expression of YKL-40 is significantly associated with higher glioma grade and poorer clinical outcome (10, 11). Stress conditions such as hypoxia, ionizing radiation, starvation, and chemotherapy have been shown to cause an increase in YKL-40 expression (12).

mAY, a neutralizing antibody that targets YKL-40, was developed as a potential therapeutic agent for GBM. It abolishes the activation of the VEGF receptor and MAPK signaling pathway, inhibits tumor growth and angiogenesis, and enhances cell sensitivity to γ-irradiation (13). In animal xenograft models, YKL-40 antibody treatment combined with ionizing irradiation dramatically inhibited tumor vascularization and progression and increased the overall survival rate (14). However, a recent study reported that dual targeting of VEGF and YKL-40 using bevacizumab and YKL-40 monoclonal antibodies did not prolong survival in glioma tumor–bearing mice (9). In addition, recent studies have demonstrated that YKL-40 may play different roles in GBM progression in the context of different MGMT promoter methylation statuses (15). Considering the diverse roles that YKL-40 plays in cancer cells, the tumor microenvironment, and the immune system (16, 17), the translational significance of targeting YKL-40 in GBM needs to be investigated further.

In addition to the extensively studied full-length YKL-40, a short variant generated by alternative splicing has also been identified in human cells, although the function of this short isoform is also unknown (18). In this study, we examined the expression of the short isoform in a panel of GBM cell lines, cloned its DNA sequence, and further confirmed the existence of this short isoform in human cells. In addition, functional analysis using an overexpression model showed that the two isoforms of YKL-40 play opposing roles in terms of protein secretion, cell proliferation, and migration by differentially regulating several downstream signaling pathways. These novel findings may provide an extended understanding of the biological function of YKL-40 and suggest new ideas for anti-YKL-40–based targeted therapy.

## Materials and methods

### Cell lines and cell culture

HEK293T, A172, A207, T98G, CRL, U251MG, U373MG, A1235, and U87MG cell lines were maintained in Professor Andrew Chan’s Lab (School of Biomedical Sciences, The Chinese University of Hong Kong). All cells were cultured in Dulbecco’s modified Eagle’s medium (DMEM) (Gibco, 12800-017), supplemented with 10% fetal bovine serum (FBS) (Gibco, 16000044) and Pen/Strep (100 mg/ml) (Gibco, 15140-122) at 37°C in 5% CO2. YKL-40–overexpressing cell lines (including U251, A172, A1235, T98G, and U87MG) were maintained in complete DMEM with 0.3 µg/ml puromycin.

### Expression vectors

Full-length YKL-40 was cloned from U87MG cells, and the short YKL-40 isoform was cloned from U251MG cells. The primers used for cloning were:

YKL-40 Fw, CGGGATCCACCATGGGTGTGAAGGCGTCTCAAACA  YKL-40 Rev, ACGCGTCGACCTACGTTGCAGCGAGTGCATCCTT   PCR products were checked by agarose gel electrophoresis and purified by a gel extraction kit (Geneaid, DF300). Purified DNA fragments were digested with *Bam*HI (NEB, #R0136T) and *Eco*RI (NEB, #R0101T) and cloned into the pBABE-puro and pBABE-Neo (Addgene, #1767) vectors using T4 DNA ligase (NEB, #M0202T). All constructs were confirmed by Sanger sequencing. Confirmed plasmids were transformed into *Escherichia coli* DH10B competent cells, and plasmid DNA used for cell transfection was extracted using an endotoxin-free plasmid kit (TIANGEN Biotech, #DP117).

### Cell lysate and immunoblotting

Cells were lysed with RIPA buffer (50 mM Tris-HCl pH 7.5, 1% Triton X-100, 150 mM NaCl, 10 mM MgCl_2_, 0.1% SDS, 0.5% sodium deoxycholate, and protease inhibitor cocktail). The total protein concentration was determined by a BCA protein assay kit (Thermo Fisher, #23225). Cell lysates were mixed with 5× Laemmli sample buffer, boiled for 4 min at 95°C, and then stored at −20°C. Total cell extracts were resolved on an SDS–PAGE gel.

The following antibodies were used for immunoblotting: YKL-40 (Abcam, #ab77528), YKL-40 (Cell Signaling, #47066), Actin (Santa Cruz, #sc-1615), anti-goat horseradish peroxidase (HRP)-conjugated secondary antibody (Santa Cruz, #SC-2352), anti-rabbit HRP-conjugated secondary antibody (Cell Signaling, #7074P2), and anti-mouse HRP-conjugated secondary antibody (Cell Signaling, #7076s).

### RNA interference

Small inhibitory RNA (siRNA) sequence pools against YKL-40 (ON-TARGETplus CHI3L1 siRNA, # L-012568-01-0005) and pooled scrambled control siRNA (ON-TARGETplus Non-Targeting Pool, # D-001810-10-05) were purchased from Dharmacon. siRNAs were transfected into U87MG and A1235 cell lines using DharmaFECT 1 transfection reagent (Dharmacon, # T-2001-01). Protein samples were collected 60 h after siRNA transfection.

### Cell proliferation assay

U251MG and A1235 cells stably expressing full-length (FL) or short variant (SV) YKL-40 were generated by retroviral infection. In total, 2.5 × 10^4^ cells were plated into 12-well plates and incubated with full serum DMEM (10% FBS) or reduced serum DMEM (1% FBS). The cells were then trypsinized into a suspension, stained with trypan blue, and counted using a hemocytometer on Days 1, 3, and 5.

### Cell migration assays

For the wound healing assay, cells were seeded into a 6-well plate and cultured until they reached > 90% confluence. A sterile pipette tip was used to scratch a linear wound, and wound healing images were captured using an inverted microscope (Nikon TI-S, Japan). The relative areas of wound closure were analyzed by ImageJ software.

Cells were resuspended in 200 μl of reduced serum DMEM (1% FBS) at a density of 2.5 × 10^5^ ml and seeded into the upper chamber (Corning, USA) for the cell migration assay. The lower chamber was filled with 700 μl of full serum DMEM (10% FBS). Transwell chambers were placed in an incubator (37°C, 5% CO2) for 16 h. Cells on the upper membrane surface were physically removed. Cells that had migrated to the lower side of the membrane were fixed with 4% paraformaldehyde for 15 min and permeabilized with 0.2% Triton X-100, and the nuclei were stained with 2 μg/μl DAPI. After each step, the samples were washed with phosphate buffered saline (PBS). Finally, the cells were counted under an inverted microscope (Nikon TI-S, Japan). Five random fields were selected for statistical analysis under a microscope and photographs were taken. All assays were repeated 3 times.

### Immunofluorescence

U87MG and U251MG cells stably expressing YKL-40 FL or SV were plated into 8-well chamber slides at a density of 8,000 cells per well. Forty-eight hours after seeding, the cells were washed twice with PBS, fixed with 4% formaldehyde solution in PBS for 15 min, and permeabilized with 0.5% Triton X-100 in PBS for 12 min. Permeabilized cells were blocked with blocking buffer (3% bovine serum albumin in PBS) for 1 hour at RT. The slides were incubated with anti-CHI3L1/YKL-40 (Abcam, #ab77528) and anti–protein disulfide isomerase (PDI) (Abcam, #ab2792) antibodies, followed by incubation with an Alexa Fluor 488-conjugated donkey anti-rabbit IgG (H + L) antibody (Thermo Fisher, #A-21206) and an Alexa Fluor 555-conjugated donkey anti-mouse IgG (H + L) antibody (Thermo Fisher, #A-31570). The nucleus was stained with 300 nM DAPI in PBS for 15 min at RT. Slides were mounted with mounting medium (VECTOR laboratories, #H-1200) and covered with large coverslips (24 mm × 50 mm). Cells were examined by confocal fluorescence microscopy (Olympus FV1200 SIM Confocal System with ZDC, Olympus). The fluorescence signals were quantified by Fiji-ImageJ software.

### Quantitative RNA sequencing and data analysis

U251MG cell lines with YKL-40 overexpression were cultured in DMEM with 10% FBS and lysed in 1 ml TRIZOL™ Reagent (Invitrogen, #15596026). Total RNA was extracted according to the manufacturer’s instructions. The RNA samples were then subjected to quantitative RNA sequencing by BGI (Beijing Genomics Institute, Shenzhen). The threshold for differentially expressed genes (DEGs) was set as log2 value >0.7 and the *p*-value was set as <0.05. The DEGs were then integrated, and the extent of overlap was presented in Venn diagrams (http://bioinformatics.psb.ugent.be/webtools/Venn/) and heatmaps (MORPHEUS, Broad Institute). For the functional analysis, a gene set enrichment analysis (GSEA) (Broad Institute) toolkit was used to obtain an enrichment score for defined gene sets, such as hallmark, GO, and KEGG pathway gene sets.

## Results

### The expression of CHI3L1/YKL-40 is upregulated in GBM with poor prognosis

**Figure 1:**
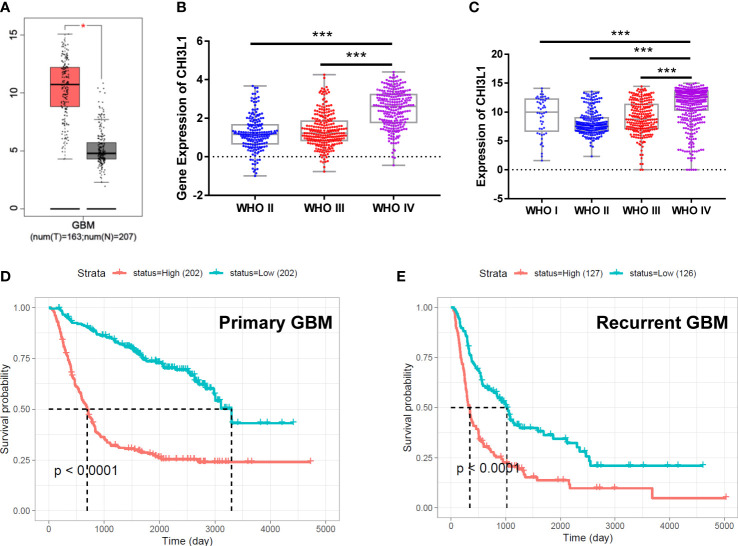
The expression of CHI3L1/YKL-40 is upregulated in GBM cases with poor prognosis. **(A)** YKL-40 expression is upregulated in GBM patients compared with normal samples based on data from the GEPIA database (tumor tissue in red box, normal tissue in gray box, *p<0.05). **(B, C)** In data from the CGGA and GENT databases, YKL-40 mRNA levels were positively associated with the histological grade of the glioma (***p<0.001) **(D, E)** Kaplan-Meier curve of the probability of survival in primary **(D)** or recurrent GBM **(E)** by difference in the expression of YKL-40, based on data adopted from the CGGA database.

GBM frequently features increased expression of CHI3L1/YKL-40, which in turn correlates with poor prognosis (19). We analyzed the expression of YKL-40 in GBM transcriptome data from 163 GBM cases and 207 nontumor brain tissues using Gene Expression Profiling Interactive Analysis (GEPIA). The mRNA levels of CHI3L1/YKL-40 were significantly upregulated in GBM tumor tissue compared to normal tissue **(** **Figure 1A** **)**. The expression of CHI3L1/YKL-40 was also positively associated with the histological grade of the glioma, based on data from the Chinese Glioma Genome Atlas (CGGA) and the Gene Expression Database of Normal and Tumor Tissues (GENT) **(** **Figures 1B, C** **)**. To further validate the clinical significance of YKL-40 in GBM, we evaluated the association of CHI3L1/YKL-40 expression levels with clinicopathological features in the CGGA database. Kaplan-Meier survival analysis showed that high CHI3L1 mRNA levels were significantly associated with poor prognosis in both primary and recurrent GBM cases **(** **Figures 1D, E** **)**. However, a similar trend was not observed in the TCGA dataset (**Figure S1A**). This discrepancy may reflect the generally poor prognosis associated with GBM, with most patients being diagnosed at a very late stage and harboring high YKL-40 expression levels. Further analysis using the 675 cases from the TCGA-LGG (low-grade glioma) dataset showed a similar trend to the CGGA datasets (**Figure S1B**), indicating that aberrant expression of YKL-40 strongly influences lower stages of glioma other than GBM.

### Identification of a short variant of CHI3L1/YKL-40

**Figure 2:**
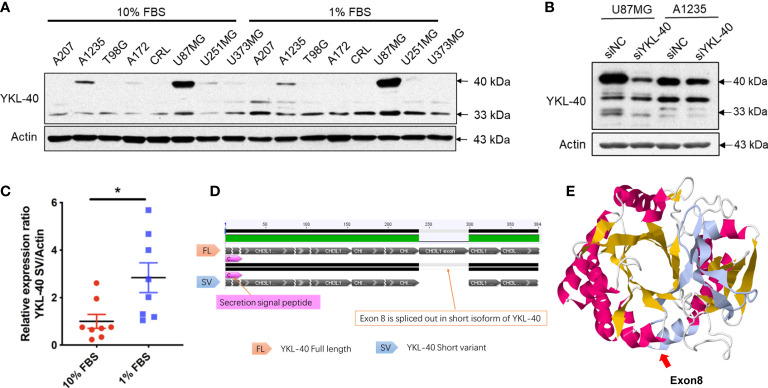
Expression patterns of YKL-40 isoforms in GBM cell lines. **(A)** Eight GBM cell lines cultured in full serum DMEM (10% FBS) or reduced serum DMEM (1% FBS) were lysed with RIPA buffer, and the expression levels of YKL-40 were determined by Western blot analysis. **(B)** U87MG and A1235 cells transfected with either siYKL-40 or siNC were lysed with RIPA buffer, and the expression levels of YKL-40 were determined by Western blot analysis. **(C)** The expression of the YKL-40 short isoform was significantly upregulated after low-serum treatment (Bars, SEM. Student’s paired *t*-test. *p < 0.05, *n* = 8). **(D)** Pairwise alignment of the cDNA sequences of the two isoforms of YKL-40. **(E)** Stereo view of the YKL-40 protein structure. The structure of exon 8 is indicated in gray.

To explore the expression of YKL-40 in GBM, eight GBM cell lines cultured with either full-serum (10% FBS) or low-serum (1% FBS) conditions were analyzed. Western blotting revealed high levels of YKL-40 in several GBM cell lines (**Figure 2A**). Compared to normal human primary astrocytes, the expression of YKL-40 in GBM cells was dramatically upregulated (**Figure S2**). Surprisingly, in addition to the main band of YKL-40, we also noticed that the YKL-40 antibody reacted with an unidentified protein of lower molecular weight (approximately 33 kDa). This protein was expressed in a pattern different from the full length. To test whether this unknown protein was an isoform of YKL-40, siRNA targeting YKL-40 was used to knockdown the mRNA expression. The results of the Western blot analysis showed that the density of the lower bands was dramatically reduced in both U87MG and A1235 cells after siRNA transfection (**Figure 2B**). The expression of the short isoform was significantly upregulated after low-serum treatment (**Figure 2C**), but this trend was not observed in the expression of full-length YKL-40, which might indicate that these two isoforms of YKL-40 play different roles in the regulation of cellular activities in GBM. The DNA sequences of the two isoforms were cloned from cDNA extracted from U251MG cells, and pairwise sequence alignment revealed that exon 8, including 61 amino acids, was spliced out in the short isoform (**Figure 2D**). For the stereo view of the short isoform, the β-strand β7 of the (β/α)_8_ barrel and the beginning two β-strands of the secondary antiparallel β-strands (α + β domain) were missing in the crystal structure (**Figure 2E**—missing part marked in gray), which may disrupt the structure of YKL-40’s carbohydrate-binding groove.

### Distinct subcellular localization of full-length and short YKL-40 variants

**Figure 3:**
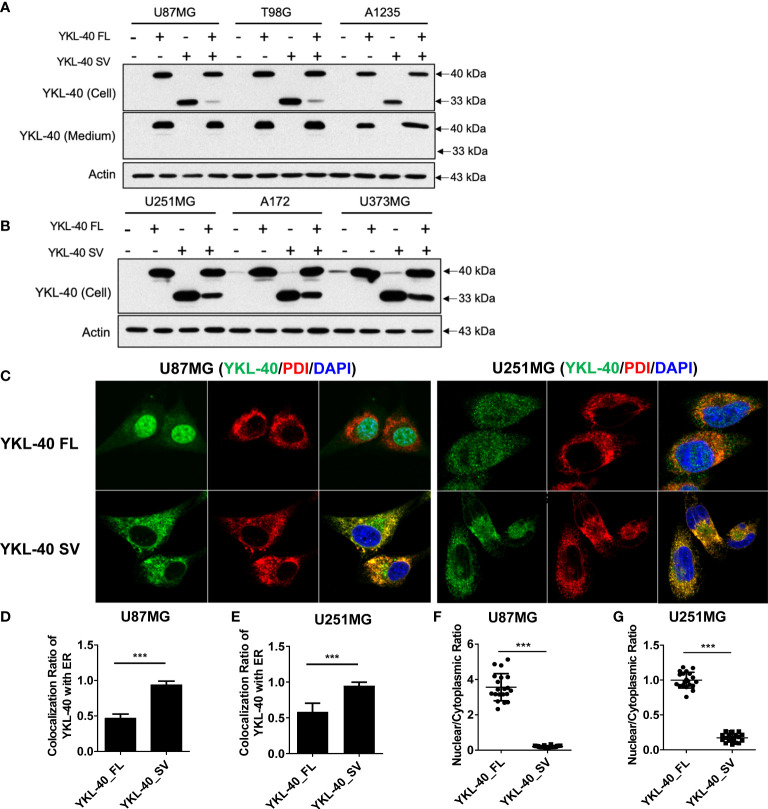
Distinct subcellular distribution of YKL-40 full-length (FL) and short variant (SV) isoforms in GBM cell lines. **(A, B)** GBM cell lines with stable overexpression of both isoforms of YKL-40 were established by retroviral transfection, and the expression and secretion of YKL-40 were detected by Western blot analysis. **(C)** U87MG and U251MG stable lines overexpressing FL and SV YKL-40 were fixed and stained with anti-YKL-40 and anti-PDI antibodies, followed by Alexa Fluor 488 (green) and Alexa Fluor 555 (red) conjugated secondary antibodies. Cell nuclei were stained with DAPI (blue) (magnification 600×). **(D, E)** The colocalization ratio of YKL-40 to the ER marker PDI was calculated using Fiji-ImageJ software with the Coloc 2 plugin. **(F, G)** The nuclear to cytoplasmic localization ratio of YKL-40 was quantified using Fiji-ImageJ. (Bars, SD. Student’s *t*-test. ***p < 0.001. The relative intensity of YKL-40 was quantified from 15 to 25 individual cells).

To explore the biological function of short isoforms of YKL-40, GBM cell lines stably overexpressing both isoforms were generated (**Figures 3A, B** **)**. Although both YKL-40 FL and SV contain the consensus signal peptide sequence in their N-terminal regions, they differ drastically in their ability to be secreted into the extracellular compartment; only YKL-40 FL was secreted into the culture medium (**Figure 3A**). Immunofluorescence analysis showed striking differences in their staining patterns (**Figure 3C**). The YKL-40 SV protein was closely associated with the endoplasmic reticulum (ER), which stained positively with the ER marker protein disulfide isomerase (**Figures 3D, E** **)**. These results indicated that, with the structure of the carbohydrate-binding groove compromised, the SV protein failed to be transported from the ER to the Golgi compartments, which further blocked its secretion. Furthermore, YKL-40 FL displayed a prominent nuclear and cytosolic staining pattern. In contrast, YKL-40 SV was almost completely absent from the nuclear compartment and localized predominantly to the cytosol (**Figures 3F, G** **)**.

### The YKL-40 short variant inhibits GBM cell proliferation and migration

**Figure 4:**
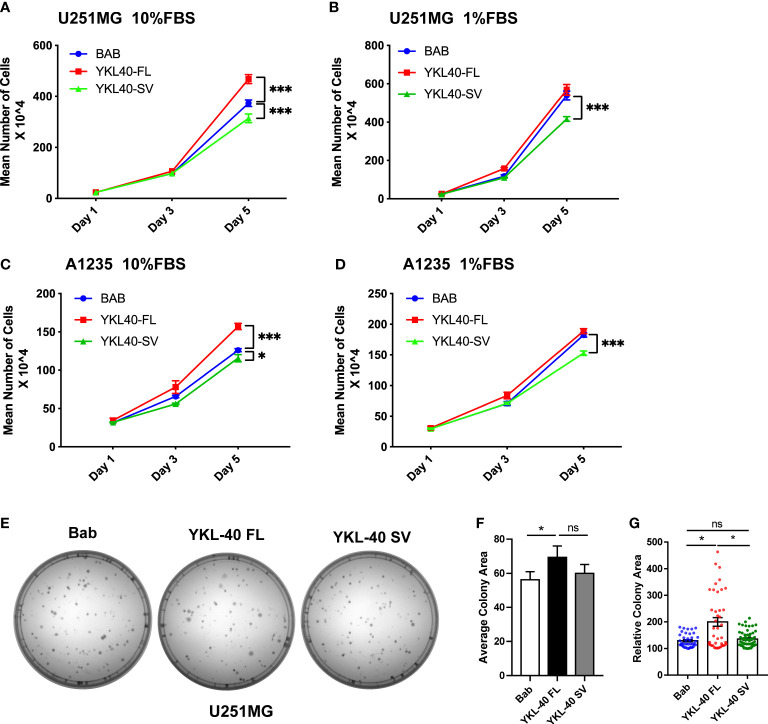
Opposing effects of YKL-40 full-length (FL) and short variant (SV) isoforms on cell proliferation. U251MG and A1235 cells were plated into 12-well plates supplemented with full-serum medium (10% FBS) **(A, C)** or reduced-serum medium (1% FBS) **(B, D)**, and counted on Days 1, 3, and 5 (Bars, SD. Two-way ANOVA. ***p < 0.001; *p < 0.05). BAB, Blank vector control; YKL40-FL, YKL-40 full-length; YKL40-SV, YKL-40 short variant. *N* = 4). **(E)** U251MG cells were seeded into 60 mm culture dishes at a density of 200 cells/plate and cultured for two weeks to form colonies. The cell colonies were stained with crystal violet. **(F, G)** Colony areas were quantified by Fiji-ImageJ software. (Bars, SD. One-way ANOVA. *p < 0.05; ns, no significant difference. *n* = 3) The relative colony area was calculated from 40 to 60 individual colonies.

Full-length YKL-40 has been reported to play an oncogenic role in various types of cancers, including GBM. Based on the distinct subcellular localization patterns of the two isoforms, we further tested the biological function of these two isoforms in GBM cell lines that express relatively low levels of YKL-40. U251MG cells overexpressing YKL-40 FL displayed greater proliferation rates of 20% and 33% compared to the controls and to cells expressing the SV isoform, respectively (**Figure 4A**). This effect was more prominent when cells were cultured in full (10% FBS) rather than in low serum (1% FBS) (**Figure 4B**). The proliferation of cells overexpressing the SV isoform was reduced by 10% compared to the control (**Figure 4A**). A similar inhibitory effect of the SV isoform was also observed in another GBM cell line, A1235 (**Figures 4C, D** **)**. Moreover, U251MG cells overexpressing SV isoforms were more sensitive to AKT inhibitor (MK2206) treatments. In contrast, FL isoform expression increased resistance to MK2206 treatments (**Figure S3A** **).** Colony-formation assays showed that overexpression of the YKL-40 FL isoform led to a dramatic increase in colony area in U251MG cells, and some large clones were also observed in the YKL-40 FL group (**Figures 4E–G**). Unfortunately, the inhibitory effect of the SV isoform was not significant in this experiment (**Figure 4G**). In addition, a cell proliferation assay was also performed in another GBM cell line, A172 (**Figure S3B**). The SV isoform also failed to inhibit cell growth, and this inconsistency may be due to heterogeneity across different GBM cell lines.

**Figure 5:**
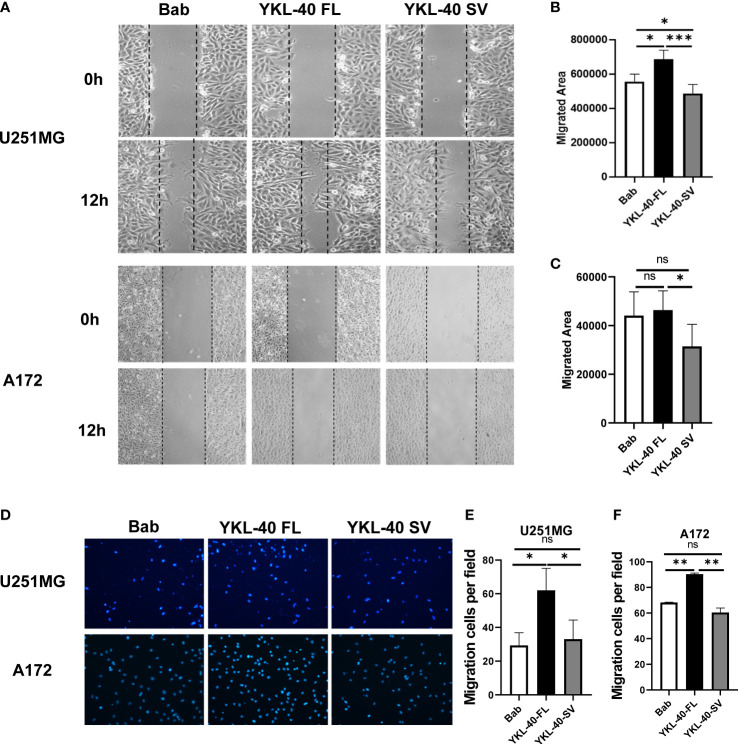
The short variant of YKL-40 significantly inhibits horizontal migration. **(A)** Monolayers of U251MG or A172 cells were scratched with a 200 μl pipette tip when they reached 95% confluence. Representative images of migrating cells were taken under an inverted microscope (magnification 40×). **(B, C)** The migration of the U251MG and A172 cells was quantified by wound closure areas 12 h after scratching (wound gap area – area after closure). (Bars, SD. One-way ANOVA. *p < 0.05; **p < 0.01; ***p < 0.001; ns, no significant difference. *n* = 6). **(D)** U251MG or A172 cells that migrated to the bottom side of the transwell chamber were fixed and stained with DAPI. Representative images of migrating cells were taken under an inverted microscope (magnification 100×). **(E, F)** The number of migrated cells was quantified by Fiji-ImageJ software. (Bars, SD. One-way ANOVA. *p < 0.05; **p < 0.01; ***p < 0.001; ns, no significant difference. *n* = 3).

To determine whether the YKL-40 SV isoform plays an inhibitory role in cell migration, wound-healing and transwell assays were performed to monitor migratory ability. Overexpression of the SV isoform impaired wound healing by 10%–20% in both cell lines compared with the cells transfected with blank vectors (**Figures 5A–C**). In terms of the vertical migration ability, the SV isoform showed no significant effect on inhibiting the transwell ability in either cell line (**Figures 5D–F**). Consistent with previous reports, the FL isoform promoted cell migration on both the horizontal and vertical axes (**Figures 5A, D** **)**.

### The two isoforms of YKL-40 display opposing roles in signaling regulation on RNA-seq analysis

**Figure 6:**
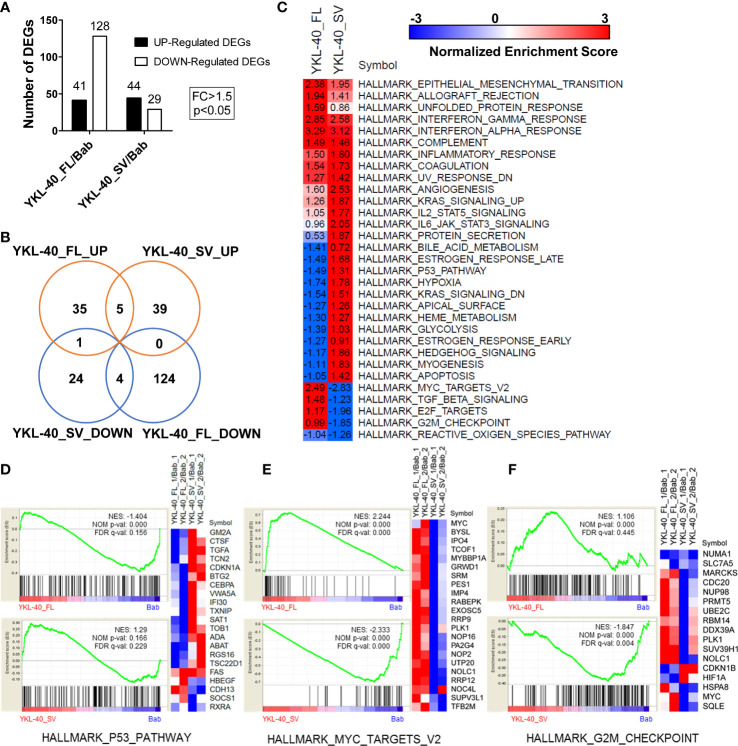
YKL-40 full-length (FL) and short variant (SV) isoforms elicit distinct transcriptional profiles in U251MG cells. **(A)** Transcriptional profiles of U251MG cells stably expressing YKL-40 FL and SV were generated by RNA-seq. Differentially expressed genes (DEGs) were identified by comparison to the control group using the following thresholding criteria: fold change > 1.5, *p*-value < 0.05. **(B)** A Venn diagram showing the common or specific genes regulated by YKL-40 FL or SV was generated by an online tool. **(C)** GSEA enrichment analysis delineated cancer-related hallmark-associated gene sets. The hallmark gene sets from the MSigDB resource were utilized in the analysis, NES, Normalized Enrichment Score; positive and negative values of NES indicated up- and downregulated gene expression patterns, respectively (FDR *q*-value <5%, *q*-value <0.05). **(D–F)** Selective hallmark gene sets showing the opposing effects of two different isoforms were generated by the GSEA tool kit. A heatmap showing the RNA expression levels (relative expression ratio compared to control) of the DEGs included in certain gene sets.

To uncover the underlying mechanisms responsible for the distinct biological properties of the YKL-40 FL and SV isoforms, RNA-seq analysis was performed in U251MG cells overexpressing these two isoforms, revealing drastic differences in their transcriptional profiles. While both FL and SV isoforms upregulated more than 40 genes, FL downregulated more than 4 times as many genes as SV (**Figure 6A**, **Supplementary Tables 1**, **2**). Strikingly, the FL- and SV-regulated genes showed very little overlap (**Figure 6B**).

Preranked GSEA using a subset of the cancer hallmark functional gene sets from the MSigDB collection was performed to identify differential regulation of pathways or biological processes by the two isoforms of YKL-40. This analysis revealed that both isoforms could alter several hallmarks related to inflammation, epithelial–mesenchymal transition, and angiogenesis (**Figure 6C**). In addition, the two isoforms showed opposing roles in regulating multiple tumorigenesis pathways. The FL isoform mostly downregulated pathways associated with tumor suppression, such as p53, anti-KRAS, and apoptosis, and upregulated oncogenic signaling targets such as MYC and E2F (**Figures 6D, E** **)**. In contrast, the SV isoform displayed completely opposite regulatory effects on these signaling pathways (**Figures 6D–F**).

## Discussion

Elevated levels of YKL-40 have been observed in many cancer types (19). YKL-40 has been found to be the most highly expressed gene in glioblastoma and in comparison to normal brain tissues (20). Altered expression of YKL-40, a characteristic of the mesenchymal GBM subtype (7), promotes GBM cell proliferation, migration, and invasion (21). In GBM patients, the expression of YKL-40 is responsible for resistance to radiotherapy (11) and TMZ chemotherapy (22). Altered expression of YKL-40 facilitates a pro-tumorigenic microenvironment composed of activated microglia and macrophage infiltration, which promotes cancer cell proliferation and migration (23). By analyzing GBM transcriptomes from different online databases, we found that YKL-40 was highly overexpressed in GBM, and that high levels of YKL-40 expression were associated with poor prognosis for both primary and recurrent GBM cases (**Figures 1A–E**).

In addition to the extensively studied YKL-40 isoform, a short isoform was also observed in our Western blot results (**Figure 2A**). This short isoform has previously been reported in muscle tissue, but its biological function is still unknown. To investigate further, we cloned the CDS region of both isoforms and established stable overexpression cell lines to explore their functions in the progression of GBM. In contrast to the secreted full-length isoform of YKL-40, the short isoform with exon 8 deletion was unable to be secreted (**Figure 3A**). The staining results showed that the short variant was retained and accumulated in ER-like structures and failed to be transported to secretory vesicles (**Figure 3C**). Since both isoforms have a signal peptide, we speculate that the long and short forms are both recruited to the lumen of the ER, but only the long form is able to be transported out of the ER and enter the Golgi apparatus to further process through the secretory pathway. The short fragment encoded by exon 8 may be essential for YKL-40 to interact with transporter proteins that are responsible for protein translocation from the ER lumen to the Golgi complex. In future studies, a coimmunoprecipitation (Co-IP) assay could be employed to enrich the binding partners of YKL-40 FL or SV, and proteins that coprecipitated with full length YKL-40 but not the short variant could be subjected to mass spectrometry analysis. Identification of the transportation partner for YKL-40 is also important for developing chemical compounds that target YKL-40 secretion.

In addition, our results revealed that the short isoform of YKL-40 played an opposing role in the regulation of cell proliferation and migration. Different isoforms of the same gene often play different or even opposing roles in the progression of carcinogenesis (24–27). The underlying mechanism of the opposing effect can differ by gene. In accordance with previous studies, we found that overexpression of the full-length YKL-40 protein significantly promoted cell proliferation, migration, and invasion in glioma (28). These types of pro-oncogenic effects are driven by several known signaling pathways, such as MAPK and PI3K, either by directly binding with receptors or by signaling regulators. However, our transcriptome data suggested that YKL-40 is involved in the inflammatory response and the epithelial–mesenchymal transition (EMT) process, which were commonly upregulated by both the FL and SV isoforms (**Figure 6C**). Indeed, as an inflammatory factor, YKL-40 secretion is mediated by proinflammatory cytokines in many types of cells (29), and its expression is significantly reduced after interferon therapy in patients with HCV-associated liver disease (30). Functionally, the altered regulation of interferon signaling contributes to the immune evasion of glioma cells as well as the maintenance of glioblastoma stem cells (31, 32). In addition, regulation of EMT by YKL-40 to promote tumor progression has been observed in many cancers, including GBM (33–36). Taken in the context of our transcriptome data, it is possible that both isoforms could generate glioma stem cells that favor microenvironments contributing to GBM aggressiveness, recurrence, and resistance to radiation and chemotherapy.

Moreover, considering the opposing biological function of these two isoforms, we observed that several tumor-suppressive pathways were inhibited by the FL isoform, including p53 signaling, the G2M checkpoint pathway, and the apoptosis pathway, while these pathways were only slightly inhibited by the SV isoform (**Figures 6C, D** **)**. The most striking difference in signaling regulated by the two isoforms was in the MYC-targeted genes (**Figure 6E**). MYC proteins are among the most studied oncogenes, and they are dysregulated in more than 50% of cancers with diverse origins (37). The hallmark gene sets of MYC targets V2 (derived from the Molecular Signatures Database) included 58 genes which were regulated by MYC in many cancers (38). Several MYC-regulated genes, including MYC itself, were upregulated by the FL isoform, while the expression of these genes was significantly reduced when the SV isoform was over-expressed. This may be the key factor responsible for the opposing roles of these two isoforms. Additional extensive study will be needed to understand the mechanisms by which these genes are differentially modulated by these two isoforms. These studies will hopefully allow us to better understand the intracellular and extracellular biological functions of YKL-40.

## Conclusion

In the present study, a short splicing isoform of YKL-40 lacking exon 8 was cloned from the GBM cell line U251MG. Unlike the full-length isoform, which can localize to the nucleus and cytoplasm and be secreted, the short isoform cannot be secreted from the cell and localizes only to the cytoplasm. The function of the SV isoform acted in opposition to that of the FL isoform, thus inhibiting cell proliferation and migration. Transcriptome analysis revealed that the opposing roles of the two isoforms may modulate several oncogenic-related pathways *via* differential regulation, including p53, the G2/M checkpoint, and MYC-related signaling. This is a key finding, as it suggests the SV isoform contributes to the formation of tumor-favored microenvironments but plays a tumor-suppressive role intracellularly. The results of this study could point the way to new drugs targeted to YKL-40 alterations and provide new ideas for the development of targeted anti-YKL-40 therapy for the treatment of GBM.

## Data availability statement

The datasets presented in this study can be found in online repositories. The names of the repositories and their accession numbers can be found in the article/**Supplementary Material**.

## Author contributions

MS and QG contributed equally to designing the study, obtaining the data, and writing the manuscript. YiW, AC, and YuW conceived of/designed the experiments. XW, WD, SS, and BY established the cell lines. GW and LT helped interpret the data. ZG and AC provided advice. QG, MS, and YuW wrote and revised the manuscript. All authors contributed to the article and approved the submitted version.

## Funding

This work was supported by the Shandong Provincial Natural Science Foundation (ZR2020MH146, ZR201807090175) and a General Research Fund grant (2019WS597) from the Health Commission of Shandong Province.

## Conflict of interest

The authors declare that the research was conducted in the absence of any commercial or financial relationships that could be construed as a potential conflict of interest.

## Publisher’s note

All claims expressed in this article are solely those of the authors and do not necessarily represent those of their affiliated organizations, or those of the publisher, the editors and the reviewers. Any product that may be evaluated in this article, or claim that may be made by its manufacturer, is not guaranteed or endorsed by the publisher.
