# Supplementary material for: Functional analysis of the short splicing variant encoded by CHI3L1/YKL-40 in glioblastoma
Source: Front Oncol. 2022 Nov 2;12:910728. doi: 10.3389/fonc.2022.910728 (PMC9666495; doi:10.3389/fonc.2022.910728)
Supplement: Supplementary file 1 [file DataSheet_1.pdf]

Supplementary Material

1 Supplementary Table

Supplementary table 1&2 has been uploaded separately on submission.

2 Supplementary Figures

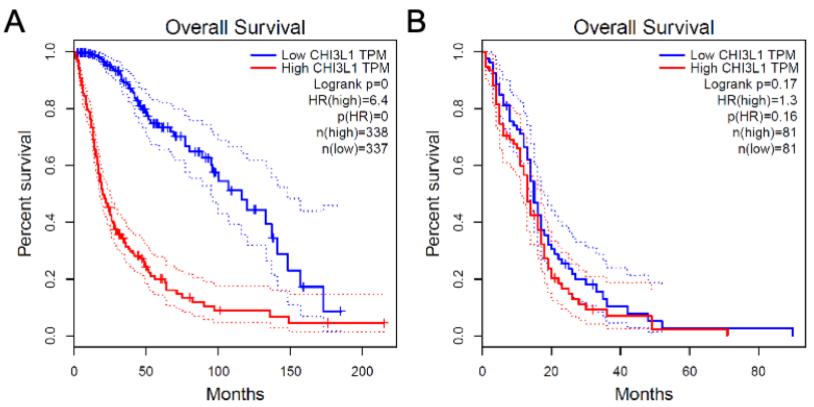

Figure S1. Kaplan–Meier curve of survival probability in LGG **(A)** or GBM **(B)** by expression difference of YKL-40, data adopted from GEPIA database.

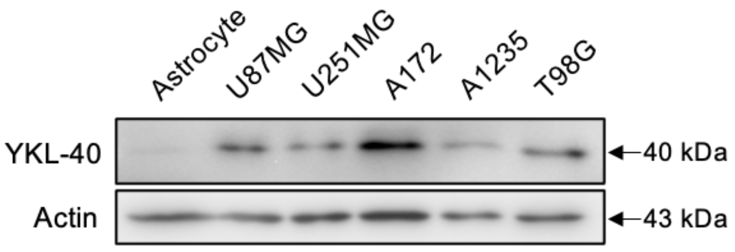

Figure S2. The expression of YKL-40 in GBM cell lines was higher than the level in primary human astrocytes.

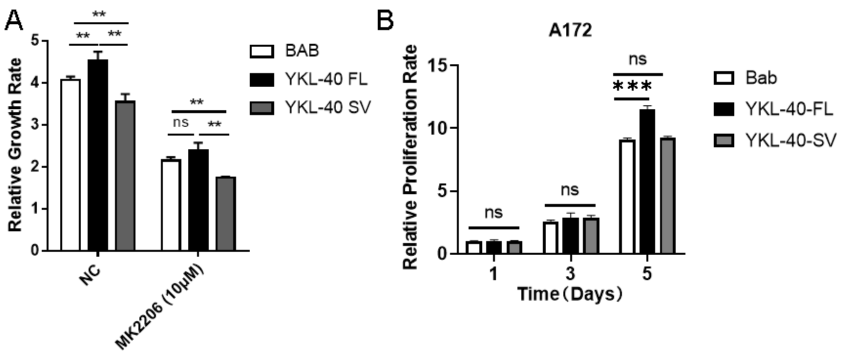

Figure S3. (A) U251MG cell was treated with MK2206 at concentration of 10 μM for 72 h, the cell viability was determined by MTS analysis. (Bars, SD. Two-way ANOVA **, p<0.01; ns, no significant difference. n=6). **(B)** A172 cell were plated in 12 well plates supplemented with full serum medium, and counted on day 1, 3 and 5. (Bars, SD. Two-way ANOVA ***, p<0.001; ns, no significant difference. n=4)
